# Think twice: Re-assessing confidence improves visual metacognition

**DOI:** 10.3758/s13414-023-02823-0

**Published:** 2023-12-22

**Authors:** Patxi Elosegi, Dobromir Rahnev, David Soto

**Affiliations:** 1 Basque Center on Cognition, Brain and Language, San Sebastian, Spain; 2 University of the Basque Country- UPV/EHU, Basque, Spain; 3 School of Psychology, Georgia Institute of Technology, Atlanta, USA; 4 Ikerbasque, Basque Foundation for Science, Bilbao, Spain

**Keywords:** Perceptual decision-making, Metacognition, Confidence calibration, Sensitivity, Change-of-mind

## Abstract

Metacognition is a fundamental feature of human behavior that has adaptive functional value. Current understanding of the factors that influence metacognition remains incomplete, and we lack protocols to improve metacognition. Here, we introduce a two-step confidence choice paradigm to test whether metacognitive performance may improve by asking subjects to reassess their initial confidence. Previous work on perceptual and mnemonic decision-making has shown that (type 1) perceptual sensitivity benefits from reassessing the primary choice, however, it is not clear whether such an effect occurs for type 2 confidence choices. To test this hypothesis, we ran two separate online experiments, in which participants completed a type 1 task followed by two consecutive confidence choices. The results of the two experiments indicated that metacognitive sensitivity improved after re-evaluation. Since post-decisional evidence accumulation following the first confidence choice is likely to be minimal, this metacognitive improvement is better accounted for by an attenuation of metacognitive noise during the process of confidence generation. Thus, here we argue that metacognitive noise may be filtered out by additional post-decisional processing, thereby improving metacognitive sensitivity. We discuss the ramifications of these findings for models of metacognition and for developing protocols to train and manipulate metacognitive processes.

## Introduction

Metacognition refers to the ability to evaluate and monitor our mental processes and actions, typically through a report of confidence. Metacognition is a fundamental feature of human cognition that can play different functional roles, such as regulating learning during early development (Taouki et al., 2022) and also in adulthood (Guggenmos et al., 2016; Hainguerlot et al., 2018), controlling information-seeking behavior and evidence accumulation prior to choice (Balsdon et al., 2020; Desender et al., 2018), and regulating the prioritization of tasks (Aguilar-Lleyda et al., 2020). Abnormal metacognition has been linked to pathological decision-making observed in various psychiatric disorders (Rouault et al., 2018). However, we do not yet understand the factors that can influence metacognitive sensitivity and we lack protocols to manipulate it in order to achieve adaptive behavior.

Previous work on perceptual and mnemonic decision-making (McLean et al., 2020; Parks & Yonelinas, 2009) has shown that type 1 perceptual sensitivity benefits from reassessing the primary choice. For instance, in a four-alternative forced choice task involving perceptual or mnemonic inputs, participants may respond incorrectly at first (choosing the wrong option out of the 4 possible ones), but if they are asked to perform a second-choice on those trials, participants are able to select the correct choice significantly above chance levels (Yeon & Rahnev, 2020). This pattern of results indicate that the remaining traces of both sensory and mnemonic evidence are in a hidden state of the system that can be re-enacted during the second choice re-evaluation. Here we ask whether a similar effect occurs for second-order confidence choices and whether re-assessing these choices improves metacognitive sensitivity. Notably, the information used to make metacognitive judgments may not be solely based on sensory information but may involve additional, post-perceptual (e.g., cognitive) processing (Pleskac & Busemeyer, 2010) or metacognitive noise reduction (Shekhar & Rahnev, 2021). Here, we introduce a second-choice confidence paradigm to test the view that metacognitive performance may be improved by asking subjects to reconsider their confidence estimates.

Most relevant for the present study is the phenomenon of change of mind in decision-making (Resulaj et al., 2009; Pleskac & Busemeyer, 2010) proposed that type 1 evidence accumulation processes may continue after a decision is made, either supporting or contradicting the initial type 1 decision. Thereby, post-decisional evidence accumulation may inform confidence choices and even lead to changes of mind (van Den Berg et al., 2016; Resulaj et al., 2009).

Although not dealing directly with confidence re-evaluation, a recent study indicates that human observers have the ability to rate their perceptual and confidence judgments in a hierarchical manner (Recht et al., 2022). Recht and colleagues used a perceptual discrimination task followed by a confidence rating to evaluate the precision of the perceptual discrimination response. Across two consecutive trials, participants were asked to provide a higher-order confidence choice by re-assessing their performance and confidence evaluations across the two trials. Finally, observers rated their certainty (i.e., either high or low) on the previous choice. Recht and colleagues (Recht et al., 2022) observed above-chance performance for higher-order confidence evaluations regarding third-order and even fourth-order ratings, thereby suggesting the existence of a higher-order (‘meta-’) metacognitive sensitivity.

Here we adopt a different paradigm to test the role of metacognitive re-evaluation. Instead of asking participants to compare their performance and confidence judgments across successive trials (Recht et al., 2022), participants were asked to re-evaluate their confidence on the same trial. Comparing confidence across trials may involve the tracking of differences in sensory signal strength or additional processes related to perceived task difficulty across trials or the attention state that is known to be taken into account in observers’ confidence ratings (Denison et al., 2018). Assessing confidence re-evaluation within the same trial is not confounded by these additional factors, as the same sensory input is used for both first- and second-confidence reports. Importantly, assessing confidence re-evaluation within the same trial allowed us to test whether or not re-evaluation plays a functional role at improving one’s metacognitive sensitivity. Evidence consistent with this hypothesis would contribute to elaborate current models of perceptual metacognition and further provide insights into the development of protocols to improve metacognition in a way that may promote adaptive behavior (Rahnev et al., 2022).

## Methods

### Transparency and openness

The experimental data and scripts are available at OSF in the following link: https://osf.io/k7uxs/?view_only=72434fce7786476ba47e4932d86c64a6. The experiments were not pre-registered.

### Participants

A total of 55 participants were recruited across two experiments from the online Prolific platform (https://www.prolific.co/). In the original experiment, 25 participants (12 women, $\overline{X}_\textrm{Age}$ = 28 years, SD_Age_ = 4.83) took part, while the replication involved a sample of 30 additional participants (13 women, $\overline{X}_\textrm{Age}$ = 27 years, SD_Age_ = 4.76). Based on the experimental results from Experiment 1, a power analysis was performed using G-Power to estimate the sample size for Experiment 2. The minimum sample size required to achieve the effect size of 0.62 reported in Experiment 1 with alpha = .05 and an expected power of 0.95 was computed. The results showed that 30 participants would be needed and accordingly we set this number for the replication study in Experiment 2. Observers were screened to ensure that they did not have any neurological or psychiatric disorders and were not taking any medication that could potentially affect their visual perception or cognitive performance. All participants reported normal or corrected-to-normal vision and had no history of color blindness or other visual impairments. For both experiments, participants provided informed consent and received monetary compensation (7.75 £/h). The study was approved by the BCBL ethics committee.

**Fig. 1:**
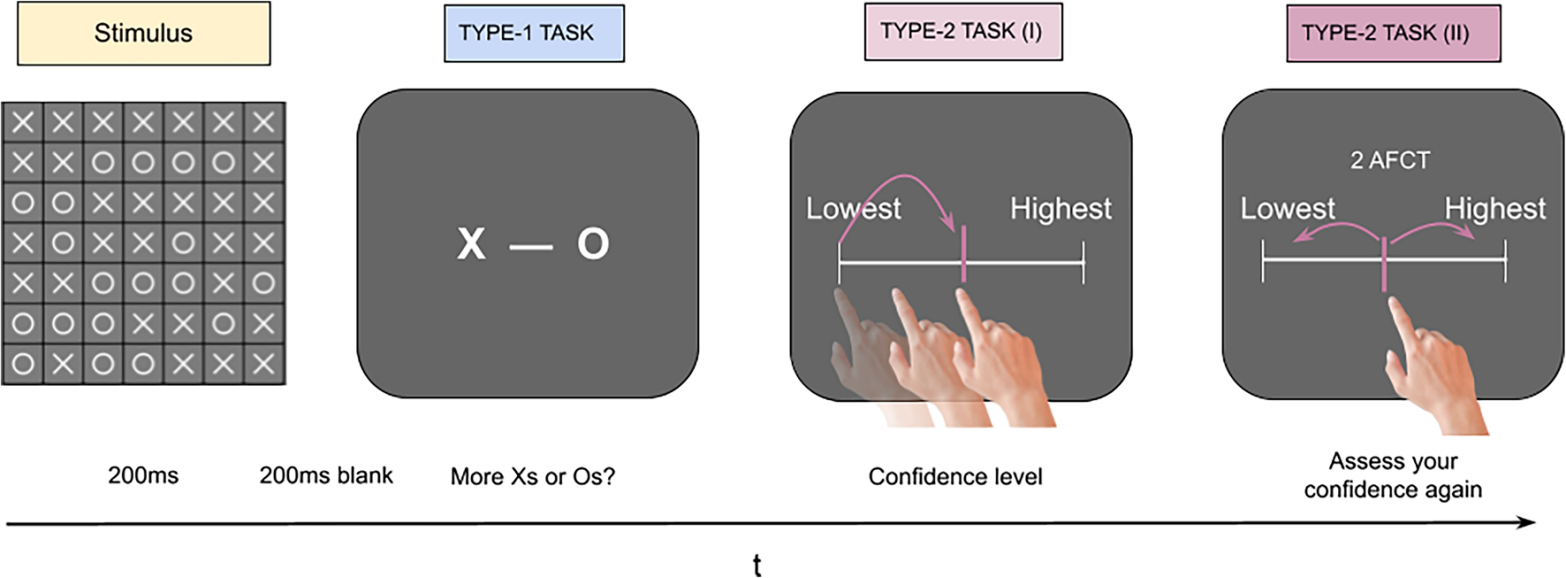
Illustration of the experimental procedure. Participants completed a perceptual task followed by two continuous confidence ratings. The stimuli consisted of two classes of shapes (Xs and Os) arranged in a 7$\times$7 grid, and participants were required to identify the predominant class in the ensemble. The stimulus was followed by a 200-ms blank before the type 1 response to avoid after image effects. Confidence was given on a 0–100 scale with step sizes of 10, and the second confidence was not allowed to be the same as the first confidence. An extreme confidence response in the first interval automatically set the end of the trial without the chance for confidence reassessment

### Experimental procedure

Figure 1 illustrates the experimental procedure, which involved a perceptual task followed by two continuous confidence ratings. The stimuli consisted of two classes of shapes (Xs and Os) arranged in a 7$\times$7 grid, and participants were required to identify the predominant class in the ensemble (Haddara & Rahnev, 2022). To ensure that the task remained challenging but avoiding floor effects, a two-up-one-down adaptive staircase adjusted the Xs and Os ratio, aiming to keep participants’ performance around 70%. Rahnev and Fleming 2019 showed that using a staircase leads to overall inflated metacognitive scores (i.e., meta-d’ or type 2 ROC). However, this is not relevant to the present study because the raw meta-d’ scores are not of interest. Given that the first and second confidence choices are given based on stimulus, any effect due to the staircase would equally affect both confidence choices. To measure type 2 performance, participants used a confidence scale ranging from *“I am not confident at all”* to *“I am completely sure that I was correct”*, with ten possible ratings. In the first confidence response, the pointer appeared randomly in one of the ten possible locations, encouraging participants to use the full range of confidence responses. In the second confidence response, the pointer reappeared in the same location as the initial response, and participants rated their confidence again. Under these circumstances, the second confidence choice can be regarded as a discrimination task in which observers can only improve or worsen their initial confidence estimates, hence allowing the computation of a second-step meta-d’ on top of the first-step meta-d’. Importantly, the same responses were not allowed in both confidence intervals. The experiment was programmed so that initial extreme confidence responses automatically set the end of the trial without the possibility for re-evaluation. Observers completed a total of 450 trials separated in nine blocks of 50 trials each and they were encouraged to take short breaks between blocks. Participants were encouraged to be as accurate as possible in both type 1 and type 2 tasks without a response deadline. Reaction times were not recorded. The experiment and the stimuli were programmed in JavaScript using OpenSesame v3.3 stimulus presentation software (Mathôt et al., 2012).

### Analysis

To maximize the quality of the data, we only considered participants who were within the range of 2.5 standard deviations from the average group-level type 1 performance. This led to the exclusion of a single participant from Experiment 1 due to a type 1 performance that fell 2.5 standard deviations below the group average. No participants from Experiment 2 met this criterion. Additionally, in order to compute signal detection theory (SDT) measures on the second interval, participants who had less than 50 *up-* or *down-shifts* trials (i.e., trials in which they increased/decreased their confidence in the second interval in relation to the first interval) were also rejected from the experiment. Consequently, the second exclusion criterion resulted in the removal of seven participants from Experiment 1 and seven participants from Experiment 2, resulting in a final count of 17 participants for the former and 23 participants for the latter experiment, respectively. These participants gave extreme first ratings likely reflecting the use of a polarized and thus less costly strategy in reporting confidence, which could be due for instance to reduced motivation.

**Fig. 2:**
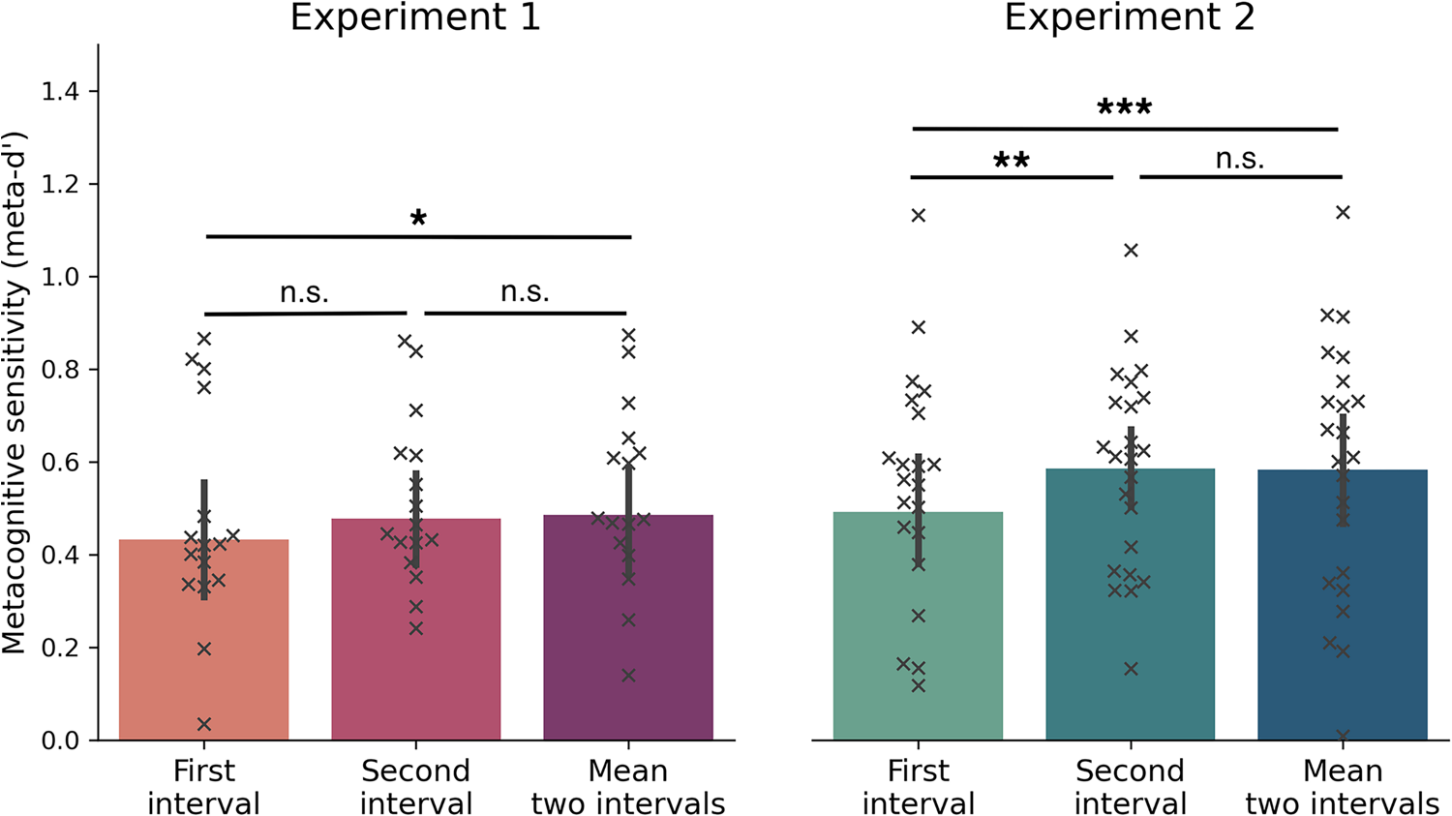
Metacognitive sensitivity (meta-d’) across the first and second intervals of confidence generation and also based on the mean confidence across both intervals. *Error bars* indicate 95% CIs, whereas *crosses* represent individual participants ($$*: p$$ < .05, $$**: p$$ < .01, $$***: p$$ < .001.)

To assess whether confidence reassessment provides an advantage to human observers, we measured metacognitive performance in three different ways. First, we calculated the meta-d’ following (Maniscalco & Lau, 2012) method, which provides a measure of metacognitive sensitivity that is independent of criterion biases. We calculated meta-d’for each confidence interval independently, as well as for the average of the two interval confidence responses. We compared these measures to determine if the combined meta-d’was greater than the first interval meta-d’, which would suggest a specific second interval advantage. Second, we also compared type 1 sensitivity between up-shift trials and down-shift trials to assess how well confidence choices track type 1 performance. If up-and down-shifts predict type 1 sensitivity, this would indicate greater metacognitive sensitivity for the second interval. Finally, we used the second confidence choices to compute a higher-order metacognitive sensitivity measure. Specifically, hits/false alarms were defined as a correct/incorrect type 1 response followed by an increase in confidence on the second interval relative to the first interval. If the second-step metacognitive sensitivity is better than the empirical chance-level estimated by shuffling the type 1 responses, that would mean that there is still some additional metacognitive sensitivity that participants can gain after the initial confidence judgment. Then, to calculate second-step metacognitive sensitivity we used the conventional d’ formula.

1 $$\begin{aligned} d' = \Phi ^{-1}(HR) - \Phi ^{-1}(FAR) \end{aligned}$$

Additionally, we also calculated the criterion in the second-step metacognitive judgment in order to quantify any possible bias towards up- or down-shifts in the second confidence choice .

2 $$\begin{aligned} c = -\frac{1}{2}(\Phi ^{-1}(HR) + \Phi ^{-1}(FAR)) \end{aligned}$$

## Results

On average, participants gave $85 \pm 54$ extreme confidence trials in the first experiment (18.88% of the total trials) and $95 \pm 73$ (21.11%) in the second experiment. As outlined in the Methods section, these trials were excluded from the analysis because they lacked a second confidence choice. This procedure yielded an average of $186 \pm 77$ down-shift trials and $179 \pm 72$ up-shift trials in Experiment 1, as well as $182 \pm 67$ of down-shift trials and $173 \pm 66$ up-shifts trials in Experiment 2. Notably, there were no statistically significant differences in the counts of up- and down-shift trials in either experiment, highlighting a well-maintained balance between both trial types (*Experiment 1*: *t*(16) = .21 , *p* = .83 , Cohen’s *d* = .10 ; *Experiment 2*: *t*(22) = .35 , *p* = .72 , Cohen’s *d* = .12). The two-up-one-down adaptive staircase method was found to be effective in titrating the ratio of Xs and Os to achieve the desired level of uncertainty in the task. Participants’ type 1 accuracy remained stable on average at around $0.65 \pm 0.01$ in Experiment 1 and $0.67 \pm 0.02$ in Experiment 2. On average, the staircase algorithm identified a ratio of 27:22 for achieving this target performance in both experiments. Further, there were no differences in the probability of up and down-shifts in the second confidence choice (*Experiment 1*: *t*(16) = .19 , *p* = .84 , Cohen’s *d* = .04 ; *Experiment 2*: *t*(22) = .36 , *p* = .72 , Cohen’s *d* = .075).

**Fig. 3:**
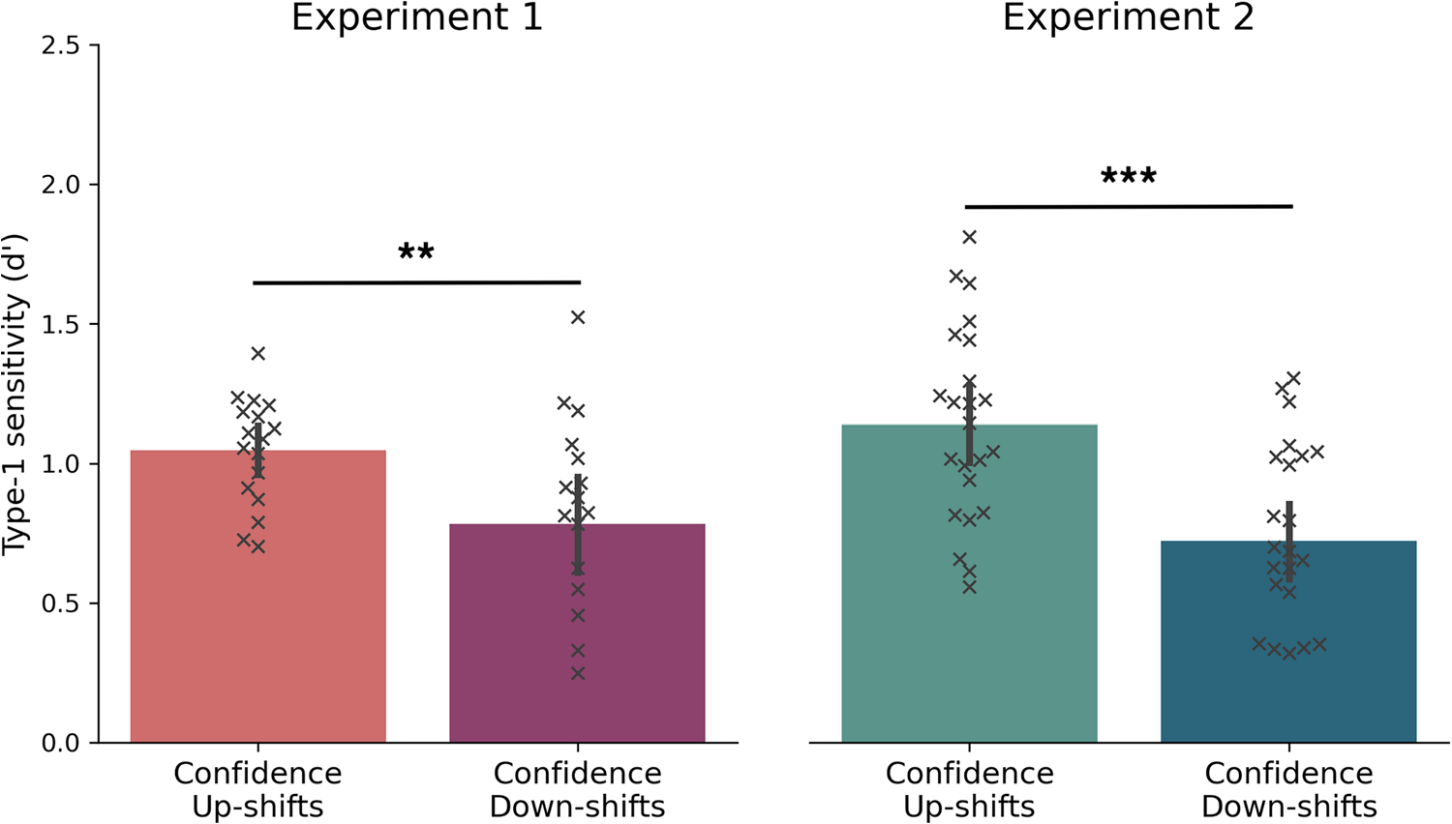
Type 1 perceptual sensitivity as a function of whether confidence increased (up-shift) or decreased (down-shift) from the first to the second confidence choice. *Error bars* indicate 95% CIs, whereas crosses represent individual participants ($$*: p$$ < .05, $$**: p$$ < .01, $$***: p$$ < .001.)

*Averaging both confidence intervals improved* *metacognitive sensitivity*. Paired-sample *t* test revealed that in the main experiment there was no difference between the first- and second-interval’s meta-d’ (*t*(16) = 1.34, *p* = .19, Cohen’s *d* = - 0.32). However, when we averaged confidence ratings across both intervals, we found that the combined meta-d’ was significantly greater than the first interval meta-d’ (*t*(16) = - 2.58, *p* = .02, Cohen’s *d* = -0.62), but not the second interval meta-d’ (*t*(16) = - 0.27, *p* = .78, Cohen’s *d* = - 0.06) (see Fig. 2 - Experiment 1). The replication study, which had a larger sample size, found significant differences between the meta-d’ values of the first and second intervals (see Fig. 2 - Experiment 2). Specifically, both the combined meta-d’ (*t*(22) = - 4.04, *p* < .001, Cohen’s *d* = - 0.84) and the second interval meta-d’ (*t*(22) = - 3.10, *p* = .005, Cohen’s *d* = - 0.64) were significantly greater than the first interval meta-d’. However, there was no significant difference between the combined and second interval meta-d’ values (*t*(22) = .142, *p* =.88, Cohen’s *d* = .03). These results suggest that there is a metacognitive improvement associated with the second interval in the main experiment, but the effect may have been too small to detect with the smaller sample size. The replication study provided stronger evidence for a significant improvement in metacognitive sensitivity associated with the second interval.

*Second-interval confidence shifts tracked type 1* *performance*. To examine whether confidence reassessment enhances metacognitive performance, we analyzed the type 1 sensitivity of observers on trials where they either increased (up-shift) or decreased (down-shift) their confidence in the second interval relative to the first interval. Since metacognitive performance is an indicator of how well confidence ratings track type 1 performance, we expected to find differences in type 1 sensitivity between the two trial types. Our results showed that in both experiments, type 1 sensitivity was significantly greater for up-shift trials than down-shift trials (*Experiment 1*: *t*(16)= 2.74, *p* = .007, Cohen’s *d* = .667 ; *Experiment 2*: *t*(22) = 5.42, *p* < .001, Cohen’s *d* = 1.13), providing support for the hypothesis that second-interval confidence shifts effectively tracked type 1 performance (see Fig. 3).

**Fig. 4:**
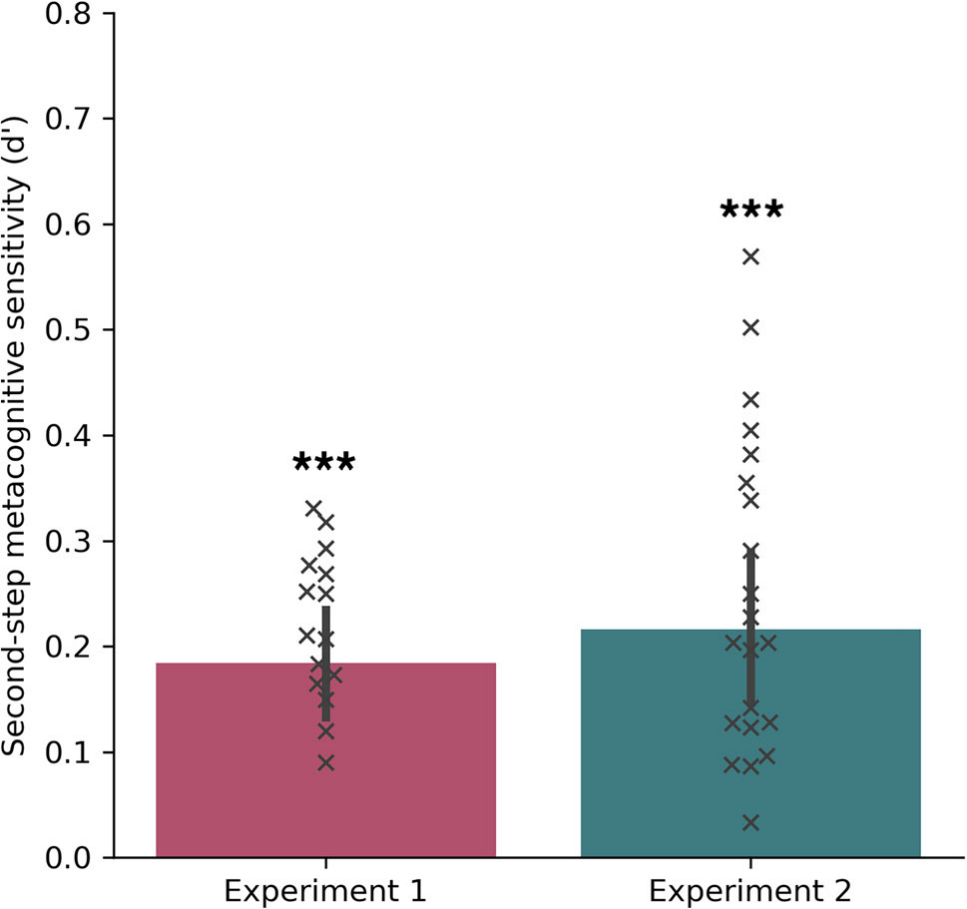
Second-step metacognitive sensitivity in both experiments. *Error bars* indicate 95% CIs, whereas *crosses* represent individual participants ($$*: p$$ < .05, $$**: p$$ < .01, $$***: p$$ < .001.)

*Second-step metacognitive performance was better* *than chance*. We calculated a higher-order metacognitive sensitivity measure by treating the up/down shifts of the second interval as a metacognitive discrimination task regarding the initial confidence (i.e., hits/false alarms were defined as an increase in confidence in the second choice on correct/incorrect trials, see Methods). We conducted paired-sample *t* tests to compare the second-step metacognitive sensitivity to empirical chance-level performance in both Experiments 1 and 2. Results showed that the second-step metacognitive sensitivity was significantly better than chance-level in both Experiment 1 (*t*(16) = 6.12, *p*<.001, Cohen’s *d* = 1.48) and the replication study (*t*(22) = 5.92, *p*<.001, Cohen’s *d* = 1.23) (see Fig. 4). This pattern of results suggest that metacognitive sensitivity improved following re-evaluation in the second confidence choice. Further, the criterion in the second confidence choice was no different from zero (*Experiment 1*: *t*(16) = .056, *p* = .95, Cohen’s *d* =.014 ; *Experiment 2*: t(22) = - .04 , *p* = .96 , Cohen’s *d* = .009), mirroring the observation of similar probabilities of up- and down-confidence shifts in the second step.

## Discussion

The present study investigated whether allowing observers to reassess their confidence leads to an improvement in metacognitive performance. Results showed that averaging both confidence intervals improved metacognitive sensitivity, with a significant increase observed in the combined meta-d’ in the main experiment and both the averaged meta-d’ and the second interval meta-d’ in the replication study. The second-interval confidence shifts were found to track type 1 performance, with type 1 sensitivity being significantly greater for up-shift trials than down-shift trials in both experiments. Additionally, the second-step metacognitive performance was better than chance, indicating a significant improvement in metacognitive sensitivity associated with the second interval. These findings provide support for the hypothesis that allowing observers to reassess their confidence leads to an improvement in metacognitive performance.

In the present study, we did not allow participants to give the same confidence in both confidence intervals in order to maximize the chances of detecting the effect of confidence reassessment on metacognition. Note, however, that it is very unlikely that participants gave an initial ‘fake’ confidence rating because the metacognitive sensitivity for first confidence ratings showed that they were significantly diagnostic of perceptual accuracy. Nevertheless, future studies should further assess the pros and cons of allowing participants to give the same confidence consecutively, and examine how rewards based on the calibration of the first vs. second confidence choices influence metacognitive sensitivity.

As noted in the Introduction, (Recht et al., 2022) showed evidence observers can estimate the reliability of confidence judgments given across two different trials of a perceptual discrimination task. This work did not deal with re-evaluation of confidence choices in single trials, as we did here, but the results are relevant in that they demonstrate the hierarchical nature of metacognitive judgments and the possibility of higher-order ‘meta’-metacognitive monitoring processes operating hierarchically. However, this perspective has been challenged by a recent report from Zheng and colleagues (Zheng et al., 2023) providing some evidence consistent with the view that such ‘meta’-metacognitive processes may not necessarily reflect hierarchical metacognitive monitoring systems. The study investigated participants’ confidence in their decision-making. Zheng and colleagues asked participants to provide three responses on the same trial (i) type 1 perceptual discrimination (ii) type 2 confidence with 2 levels (iii) type 3 confidence with another 2 levels regarding their confidence in their type 2 decision. While Zheng et al. found evidence for ‘meta’-metacognitive ability in line with Recht et al. (2022), a control experimental condition in which participants did the same perceptual task but only provided a single confidence rating on four-point scale demonstrated similar results to when participants gave two sequential confidence ratings (type 2 and type 3). Zheng and colleagues suggested that there may not be a clear distinction between type 2 and type 3 monitoring systems, and perhaps between type 1 and type 2 systems.

It may be argued that reported effects are due to post-decision evidence accumulation. Post-decision evidence accumulation has been shown even for brief masked stimuli (Rabbitt & Vyas, 1981) and here the stimulus was not masked. However, the paradigm departs in several ways from the paradigms used to support arguments for post-decision evidence accumulation. Note that here the target stimulus is unmasked and observers give a type 1 perceptual decision and then provide an initial confidence choice. To the best of our knowledge, there is no empirical ground to suggest that post-decision evidence accumulation can still linger during the second confidence choice. However, while evidence accumulation seems unlikely after type 1 and type 2 decisions, we cannot decisively conclude that it is impossible. Additional work is needed to make further determinations.

In the meantime, we are inclined to align our findings with a dynamic view of metacognition in which its efficiency increases with re-evaluation and deliberation time. It may be argued that the effect of confidence re-evaluation on metacognitive sensitivity is simply mediated by the extended timeframe for choice that is inherent to re-evaluation. Herregods et al. (2023) showed that metacognitive sensitivity measured by the area under the type 2 curve relating confidence and accuracy was higher when participants were instructed to make deliberate versus fast confidence ratings (see also Yu et al. 2015).

However, any additional accumulation of evidence about the sensory stimulus following the first confidence choice is likely to be minimal. Another, not mutually exclusive possibility, is that metacognitive noise is decreased during re-evaluation. Such filtering out may work on two different scales. First, the computational noise associated with the first and second confidence judgments may be at least partially independent. This would mean that the average confidence across the two confidence judgments would have less noise than either confidence judgement in isolation. Indeed, one of the strongest effects we observed was that the average confidence judgement was more informative than the first confidence judgement, thus providing at least partial support for partially independent noise sources in the first and second judgments. Second, our second-step metacognitive performance analyses (see Fig. 4) suggested that the second confidence is specifically more informative than the first. This finding may involve a different type of noise reduction where the second confidence is associated with smaller computational noise than the first judgement (Shekhar & Rahnev, 2021). For example, one source of computational noise for confidence judgments is confidence leak – the tendency for confidence judgments to be autocorrelated (Rahnev et al., 2015). It is possible that the extra time that elapses between the first and second confidence judgments reduces confidence leak and other similar sources of computational noise. Future research would need to disambiguate between these different possible explanations for our findings.

The work has implications for developing protocols to train and manipulate metacognition. Previous research that aimed to improve metacognitive sensitivity focused on providing feedback to participants about the calibration of their confidence judgements (Carpenter et al. 2019; but see Rouy et al. 2022, for a failure to replicate). Our results suggest that in addition to external feedback, teaching participants to control the noise arising during confidence generation, by giving participants the option of re-evaluation, may be a potential avenue to improve metacognition. For instance, computer-based tutoring systems have emerged as valuable tools for fostering metacognitive skills among learners in applied environments (i.e., classrooms) (Aleven & Koedinger, 2002; Azevedo, 2005; Carlon & Cross, 2020). These systems can incorporate confidence reassessment procedures, to further train students’ self-awareness and self-regulation skills.

## Data Availability

The authors have no known conflict of interests to disclose. The authors confirm that the data and code supporting the findings of this study are available at OSF in the following link: https://osf.io/k7uxs/?view_only=72434fce7786476ba47e4932d86c64a6. Correspondence to: p.elosegi@bcbl.eu, d.soto@bcbl.eu, Basque Center on Cognition, Brain and Language, Paseo Mikeletegi 69, 2nd Floor 20009 San Sebastian

## Open Practices Statement

The experimental data and scripts are available at OSF in the following link: https://osf.io/k7uxs/?view_only=72434fce7786476ba47e4932d86c64a6. The experiments were not pre-registered.
